# Dutch advances in cardiology: a comprehensive pci registry, telemonitoring, and graft failure analysis

**DOI:** 10.1007/s12471-025-01938-3

**Published:** 2025-02-21

**Authors:** Pim van der Harst

**Affiliations:** Department of Cardiology, Division of Heart and Lungs, University Medical Centre Utrecht, Utrecht, The Netherlands

In this issue of the *Netherlands Heart Journal* three studies highlight Dutch progress on developing personalised approaches, digital innovations, and evaluating early graft failure.

Van Geuns et al. describe the design and rationale of the South East Netherlands Heart Registry (ZON-HR) [1]. This multicentre registry captures more detailed baseline and follow-up data on patients undergoing PCI than is currently available in the NHR. Its strength lies in the focus on personalised secondary prevention—collecting comprehensive information on antiplatelet strategies, lipid management, and comorbidities such as diabetes. By identifying gaps in practice, the registry is designed to improve care and serve as a basis for future randomized trials.

The study by Van Oort et al. examines symptomatic early coronary graft [2]. Their analysis of post-CABG coronary angiographies provides an angiographic definition of early graft failure and highlights key predictors such as venous graft integration, Y‑graft configuration, and prolonged inotropic support. Nearly 60% of post-CABG angiography patients showed early graft failure, experiencing higher rates of major adverse cardiovascular events over 33 months. Despite limitations from the retrospective design and sample size, findings emphasize improved postoperative monitoring and potential surgical technique refinements.

The study by Reijrink-de Boer et al. presents data on their initial experience with a virtual atrial fibrillation (AF) clinic for patients after pulmonary vein isolation (PVI) [3]. By using smartphone-based photoplethysmography (PPG), patients can monitor their heart rhythm at home, with data sent to a central platform where trained eNurses review it. This telemonitoring approach detects AF recurrences earlier and reduces hospital visits, ECGs, and Holter recordings. High patient satisfaction highlights how digital health tools empower patients and reduce healthcare burdens in today’s fast-paced environment.

I congratulate the authors on their outstanding contributions, reinforce the importance of personalised care and innovations leading to better clinical outcomes. It is through these collaborative and forward-thinking efforts that we can continue to push the boundaries of cardiovascular medicine and offer our patients the highest standard of care.
